# Prenatal diagnosis of achondroplasia in primary care settings - Recognising the soft markers: A case report

**DOI:** 10.51866/cr.698

**Published:** 2024-11-30

**Authors:** Mohd Fudzi Wan Fadzleen Ezyani, Lili Husniati Yaacob, Razlina Abdul Rahman, Chiew Chea Lau

**Affiliations:** 1 MBBS, MMed (Family Medicine), Department of Family Medicine, Universiti Sains Malaysia, Kubang Kerian, Kelantan, Malaysia. Email: husniati@usm.my; 2 MD, Department of Family Medicine, Universiti Sains Malaysia, Kubang Kerian, Kelantan, Malaysia.; 3 MBBS, MMed (Family Medicine), Department of Family Medicine, Universiti Sains Malaysia, Kubang Kerian, Kelantan, Malaysia.; 4 MBBS, MMed (Radiology), Department of Radiology, Universiti Sains Malaysia, Kubang Kerian, Kelantan, Malaysia.

**Keywords:** Prenatal diagnosis, Achondroplasia, Primary care

## Abstract

Achondroplasia, a genetic disorder causing limb shortening, is the most common form of disproportionate dwarfism. It can be diagnosed prenatally through sonographic findings and postnatally through clinical and radiological findings. Currently, an increasing number of affected foetuses are diagnosed antenatally since prenatal ultrasonography is routinely conducted in primary care settings. Herein, we present the case of a healthy 26-year-old primigravida who received a diagnosis of achondroplasia for her foetus during the late third trimester based on her prenatal ultrasonographic findings. Following birth, the diagnosis was confirmed by the baby’s clinical and radiological findings, which showed shortening of the long bones. This case highlights the importance of recognising the soft markers of achondroplasia during routine third-trimester ultrasonography in primary care settings. Early diagnosis of achondroplasia is important to ensure timely referral to tertiary centres and adequate preparation of parents for the delivery of their baby.

## Introduction

Achondroplasia is a well-known cause of small stature with non-lethal skeletal dysplasia (SD) characterised by rhizomelic limb shortening. Its incidence is around 5-15 per 100,000 live births, while its prevalence worldwide is 4.73 per 100,000 people.^1,2^ Achondroplasia can be inherited via autosomal dominant genetic transfer, but in most cases, it involves spontaneous mutations in the fibroblast growth factor receptor 3 *(FGFR3)* gene.^3,4^

Clinically, babies with achondroplasia have disproportionate short stature with rhizomelic upper and lower limb shortening, macrocephaly with frontal bossing, trident hand, genu varum and lumbar lordosis.^4,5^ Achondroplasia is reliably detected postnatally through distinctive radiographic features of the cranium, chest, limbs, pelvis, and spine.^6^ However, prenatal diagnosis can be challenging for many primary care doctors, as this condition is uncommon. This case report highlights the importance of vigilant and correct interpretation of growth charting to ensure early prenatal diagnosis of this condition.

## Case presentation

We present the case of a 26-year-old primigravida from a non-consanguineous marriage, who was detected to have a foetus with a short femur ength (FL) at 36 weeks of gestation during antenatal follow-up at a health clinic. She initially presented for a booking visit at 13 weeks of gestation and reported no significant medical or surgical history. The couple had an average height and no family history of foetal anomaly or limb shortening. Other physical examination, blood investigation and ultrasonography at booking revealed normal findings.

**Figure 1:**
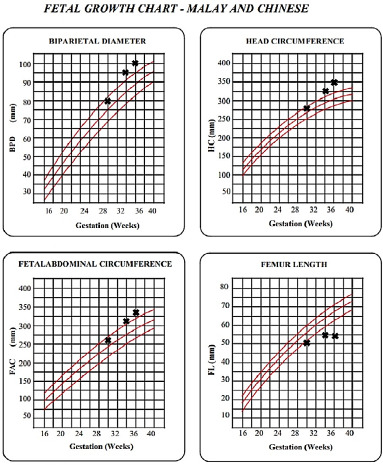
Foetal growth chart showing the femur length (FL) at the 10^th^ centile starting from 30 weeks, later plateauing and crossing the 5^th^ centile, with a relatively large head circumference (HC) and abdominal circumference (AC) (above the 90^th^ centile, with the AC increment less than the HC increment).

A subsequent detailed three-dimensional foetal scan was conducted at a private clinic at 20 weeks, showing a grossly normal foetus. Her foetus had a normal growth trend until 28 weeks of gestation. At 30 weeks, the FL was only 50 mm (10^th^ centile), but the biparietal diameter (BPD), head circumference (HC) and abdominal circumference (AC) were normal (50^th^ centile). A subsequent scan at 32 weeks revealed that the FL had fallen slightly below the 10th centile, but with normal BPD, HC and AC (50th centile). However, no action was taken at the time. The follow-up scan at 36 weeks revealed an extremely short FL corresponding to 29 weeks, while the BPD was large at the 90th centile corresponding to 40 weeks (Figure 1). The other growth parameters corresponded well to the age of gestation, with an estimated foetal weight of 2936 g. Hence, the patient was referred to the obstetrician for further investigation.

Ultrasonography by the obstetrician at 37 weeks showed a cephalic, singleton, live foetus with upper segment placenta and adequate liquor volume. All 12 long bone parameters were shortened for age, corresponding to 29 weeks. The patient was given a differential diagnosis of SD and was advised for labour induction at 38 weeks.

At 37 weeks and 6 days, she was induced with prostaglandin E2. A repeat scan showed that the foetus had a dysplastic and bowed bilateral femur and an asymmetrically enlarged BPD. The subsequent detailed scan by the MFM specialist revealed a foetus with a large head and short ribs. The humerus and femurs were bent with possible fractures, and the knee joint showed multiple cystic degenerations. The parents were counselled regarding the potential diagnosis of SD and the necessity for a gentle delivery by a skilled professional. The labour induction was terminated to proceed with an emergency caesarean section.

The mother delivered a baby girl with an excellent Apgar score of 8/10 at 1 min and 9/10 at 5 min. Her anthropometric measurements were as follows: HC of 35 cm (90th centile), weight of 3.2 kg (50th centile) and length of 45 cm (10th centile). She was noted to have a large head with frontal bossing and a depressed nasal bridge. Both the upper and lower limbs were symmetrically shortened with short fingers. The protuberant abdomen was obvious.

**Figure 2:**
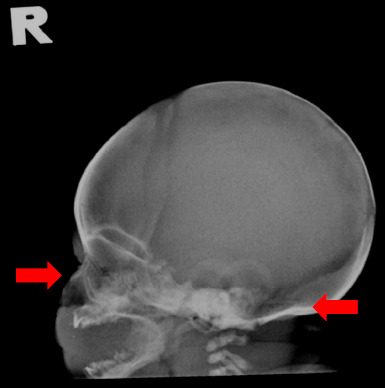
Lateral skull radiograph showing a small skull base and a depressed nasal bridge.

**Figure 3:**
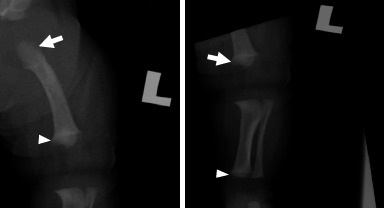
A: Left femoral radiograph showing proximal femoral lucency (arrow) and distal femoral metaphyseal cupping (arrowhead). B: Left tibial/fibular radiograph showing distal femoral metaphyseal cupping (white arrow), long fibula bone and distal tibial metaphyseal cupping (arrowhead).

The skeletal survey images favoured the diagnosis of achondroplasia. Skull radiography clearly showed a small skull base and a depressed nasal bridge (Figure 2). Other findings included flaring of the end of the anterior ribs, tombstone appearance of the bilateral iliac wings, champagne glass pelvis, trident acetabulum and proximal femoral lucency. There were also long fibula bone (Figure 3A) and metaphyseal cupping (Figure 3A and 3B). No fractures were noted.

The parents were counselled regarding the diagnosis of achondroplasia. A chromosomal study later confirmed the baby having a heterozygous mutation of the *FGFR3* gene, classified as pathogenic according to the ACGS Best Practice Guidelines for Variant Classification in Rare Disease 2020.^7^

## Discussion

Achondroplasia is a rare disease characterised by abnormal cartilage growth in the physeal regions. It is a hereditary disorder caused by a pathogenic missense variant mutation of the transmembrane portion of the *FGFR3* gene. Achondroplasia predominantly affects the growing skeleton by impairing endochondral ossification.^4^ It is the most common form of disproportionate short stature, accounting for more than 90% of cases and affects 360,00 people worldwide.^2,8^ Achondroplasia is an autosomal dominant trait.^5^ Nonetheless, the majority of reported cases are caused by spontaneous mutations, as only 20% of patients inherit it from their affected parents.^4^ In the present case, both parents had a normal adult height with no family history of dwarfism, which raised the possibility of spontaneous mutations.

Achondroplasia is usually diagnosed after birth based on clinical signs like macrocephaly, prominent forehead, midface hypoplasia, rhizomelic limb shortening, and trident hands.^1,3^ In this case, the baby exhibited many of these features, strongly suggesting achondroplasia. However, postnatal diagnosis does not allow parents time to prepare, making prenatal diagnosis the goal. Despite this, diagnosing achondroplasia prenatally remains challenging, especially in primary care settings.

In contrast to many documented cases where achondroplasia is typically identified from 20 to 24 weeks of gestation due to consistent limb shortening, this case did not display prominent markers until 36 weeks. The delay is because achondroplasia features often appear later in pregnancy, usually raising suspicion in the third trimester. Key characteristics like short FL typically emerge after 26 weeks, making it difficult for the standard 20-week anomaly scan to detect abnormalities.^4^ In this case, earlier scans showed normal FL growth, with significant shortening only apparent by 36 weeks, highlighting the diagnostic challenges in early gestation.

Once the short FL is detected, it is helpful to search for additional markers to aid in the diagnosis. A similar case report also described a case of prenatal achondroplasia diagnosed in the late third trimester, where the short FL was detected at 36 weeks.^4^ The authors also noted challenges in early detection, which delayed necessary parental counselling and preparation.

This highlights the importance of increased vigilance for SD markers during routine third-trimester ultrasonography, even without earlier anomalies.

Most primary care doctors do not have the expertise to detect many of the common skeletal defects associated with achondroplasia antenatally, such as frontal bossing, the ‘collar hoop’ sign of the bilateral proximal femur, depressed nasal bridge and narrow thorax.^9,10^ Despite limited expertise, primary care doctors may identify probable achondroplasia by measuring growth parameters and interpreting growth charts. Recent findings suggest that achondroplasia is linked to relative macrocephaly throughout gestation, with the AC rising less than the HC.^1^ In our patient, serial growth charts showed short FL with relatively large HC and AC (Figure 1), but this was overlooked, likely due to inexperience. As changes in HC and AC appear early in pregnancy, using FL, AC, and HC ratios could aid in earlier diagnosis.^1^

Given these findings, primary care practitioners should stay vigilant for achondroplasia. Early prenatal diagnosis is vital for guiding management, including referral to tertiary centres with MFM expertise for confirmation. Once confirmed, parents can receive proper counselling to prepare physically and mentally.^11^ Furthermore, recent recommendations by the European Achondroplasia Forum highlights the importance of timely genetic counselling, following a prenatal diagnosis.^12^ Genetic counselling helps families with decision-making, prognosis, and management of achondroplasia, supporting them emotionally. A multidisciplinary team of geneticists, orthopaedic surgeons, neurosurgeons, and pulmonologists is essential for anticipatory care to manage complications, improve quality of life, and promote autonomy for affected individuals.^8,12^

## Conclusion

Primary care practitioners play a vital role in identifying early signs of SD, such as discrepancies in growth parameters. Enhanced training in interpreting growth charts and recognising potential markers of achondroplasia can facilitate earlier suspicion, leading to timely referrals to MFM specialists for confirmation. This will ensure that patients and their families are better prepared mentally and physically to face the challenges associated with this rare disorder, ultimately enhancing patient care and outcomes.
